# Cellulose Acetate-Based Plasmonic Crystals for Surface-Enhanced Raman and Fluorescence Spectroscopy

**DOI:** 10.1021/acsmaterialsau.2c00013

**Published:** 2022-03-23

**Authors:** Agata Fularz, Dimitrios Stogiannis, James H. Rice

**Affiliations:** † School of Physics, University College Dublin, Belfield, Dublin 4, Ireland; ‡ Department of Physics, University of Ioannina, Ioannina 45110, Greece

**Keywords:** cellulose acetate, replica molding, imprinting, immunoassays, surface-enhanced Raman spectroscopy, surface-enhanced fluorescence

## Abstract

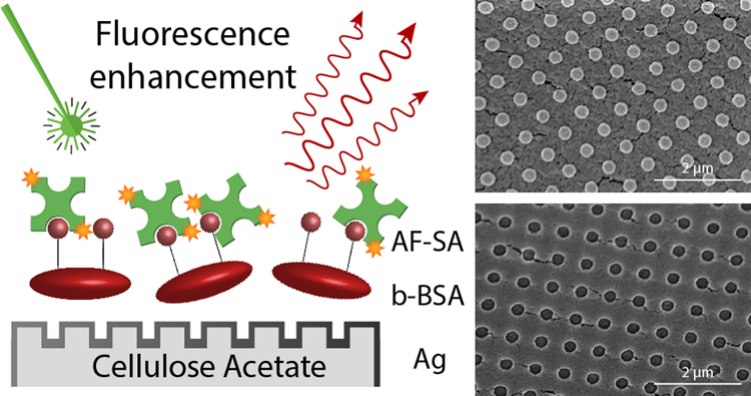

In order to meet environmental concerns, there is an increasing demand for biodegradable and sustainable materials in many areas, including photonics. Cellulose and its derivatives are potentially eco-friendly alternatives to conventional plastics, because of their abundance and lower environmental impact. Here, we report the fabrication of plasmonic structures by molding cellulose acetate into submicrometric periodic lattices, using soft lithography. The fabricated platforms can be used for the enhancement of Raman and fluorescence signals of a range of analytes including a model immunoassay utilizing a streptavidin-conjugated dye, which is characterized by a 23-fold enhancement in fluorescence signal intensity, which shows the potential of the platform to be further used for the assay-based development of diagnostic tools.

## Introduction

Detection of chemical and biological species at low concentrations is an issue of high importance in many fields including medical diagnostics,^1^ safety for explosive and chemical threat detection,^2^ and environmental analysis.^3^ Trace-molecule detection down to the single-molecule level has been previously achieved by employing spectroscopy methods, such as surface-enhanced fluorescence (SEF) and surface-enhanced Raman spectroscopy (SERS).^4−6^ Both these methods rely on the application of plasmonic substrates with features in the nanoscale, fabricated using noble metals such as silver and gold, to which selected analyte molecules can be adsorbed.^7^ These substrates enhance the light–matter interaction, resulting in stronger Raman and fluorescence signals being emitted from the sample, because of the local magnification of the electromagnetic field near the substrate, following the generation of a localized surface plasmon resonance (LSPR).^8^

To ensure high reproducibility and stability of the signal, the substrates used for Raman- and fluorescence-based sensing should be highly ordered and symmetric arrays of nanosized features such as nanoparticles or nanovoids with a specific size and lattice parameters in the order of the wavelength of the incident light, ensuring the optimal excitation of a plasmon.^9,10^ Such templates, often referred to as plasmonic crystals, generate a large number of plasmonic hotspots in the narrowly separated gaps and edges of the nanofeatures.^11,12^ Traditional methods such as electron-beam lithography^13,14^ and focused ion beam^15,16^ have been used for the fabrication of such platforms; the methods are however largely limited by the high cost, especially when it comes to the fabrication of large-area substrates for practical applications.^14,17^ More cost-effective approaches based on self-assembly, such as nanosphere lithography, are also limited, because of the lower precision and uniformity of the fabricated structures.^18,19^

An alternative approach to traditional optical lithography is soft nanoimprint lithography in which methods such as hot embossing and replica molding are used.^20^ Nanoimprinting can be used for the fabrication of uniform, large-area polymeric substrates by replicating nanopatterns from a master mold. Various polymers including polycarbonates, polydimethylsiloxane (PDMS), and polymethyl methacrylate (PMMA) among others^12,17,21,22^ have been used for the fabrication of nanostructured templates, which can then be coated with a thin layer of metal, resulting in relatively cheap, uniform plasmon-active templates that can be used for SERS and SEF-based molecule detection. The polymer used for this method, however, is of great importance, considering the recent drive to replace nonbiodegradable plastic with sustainable and biocompatible materials. Synthetic polymers are predicted to persist in landfills for millennia which combined with a low global recycling rate of about 16% is a pollution crisis with an enormous impact on ecosystems and the environment.^23^

Cellulose is the most abundant biopolymer on Earth that is present in green plants and algae and is secreted by certain types of bacteria. The polysaccharide consists of β-linked d-glucose units and can be described by the formula (C_6_H_10_O_5_)_*n*_, as shown in Figure S1a.^24,25^ Naturally occurring cellulose is composed of nanofibers with a length of several micrometers and about 3 nm in diameter,^26^ and it can be processed to obtain cellulose in the forms of nanoparticles or nanocrystals.^27,28^ Cellulose can be easily modified to obtain materials with varying structural properties, which has resulted in the material finding applications in the fields of electronics,^29,30^ energy,^31^ and sensing^32^ as well as in the fabrication of biocompatible plastic alternatives for food processing and medical use.^33,34^

Cellulose acetate (CA) is a material with the same cellulose backbone with some of the hydroxyl (−OH) groups in the structure being replaced with acetyl (CH_3_CO) groups, as shown in Figure S1b. CA is considered nontoxic, biodegradable, biocompatible, and renewable, making it a suitable material for large-scale fabrication of plasmonic platforms for biomolecule detection.^35,36^ Additionally, unlike other cellulose derivatives, CA is not water-soluble, making the material a stable platform for the detection of water pollutants and medical diagnostics.^37^

In recent years, nanopatterned cellulose-based substrates have been of great interest. The research however has focused on pristine cellulose^38,39^ and hydroxypropyl cellulose-based substrates,^20,40^ which are often limited by their high water solubility, making them unstable in humid or liquid environments.^41^ The platforms have been applied for SERS-based detection;^20,41,42^ however to our knowledge, the approach has not been expanded to SEF. Here, we show that CA can be easily patterned into flexible, nanopatterned substrates with various features such as pillars, holes, and grooves by employing the replica mouling method. The nanosized shapes are preserved after the platforms are coated with a thin layer of silver, making them suitable for SERS and SEF-based molecule detection. We show that various molecules can be detected on the substrates at concentrations as low as 10^–6^ M for Raman and 10^–7^ M for fluorescence. Additionally, we demonstrate that the substrate enhances fluorescence from a model immunoassay utilizing a streptavidin–biotin interaction, showing the potential of the approach for linking and detection of antibodies, enzymes, and other important biomolecules and the design of cost-effective substrates for fluorescence-based detection.

## Results and Discussion

The replica molding soft lithography method previously reported elsewhere^20^ was used for the preparation of imprinted CA samples. A composite stamp was prepared using hard polydimethylsiloxane (h-PDMS) and soft 184 PDMS to ensure the best uniformity and integrity of the samples.^43^ Three different designs with different shapes and sizes of nanofeatures were investigated in this study: a CA sample with grooves, prepared on a linear PDMS stamp with 417 nm period, a CA sample with rectangular posts with a lattice period of 700 nm prepared on a PDMS stamp, and a sample prepared directly on the silicon mold, resulting in rectangular holes with the same period of 700 nm. Both the lattice period and the shape of the features in a plasmonic crystal affect the excitation rate of plasmons.^44^ Localized as well as surface plasmon polaritons can be supported by the structure, with multiple plasmon modes often being generated, resulting in interaction and mixing processes.^45,46^ Because the lattice period should be on the order of the surface plasmon wavelength, molds with a lattice period close to that of the green laser light (532 nm) were chosen for the experiment. Using both the PDMS stamp and the Si mold allowed us to investigate the effect of the shape of the imprinted structures because the pillar and hole structures fabricated in such a fashion are the inverse of one another.

**Figure 1:**
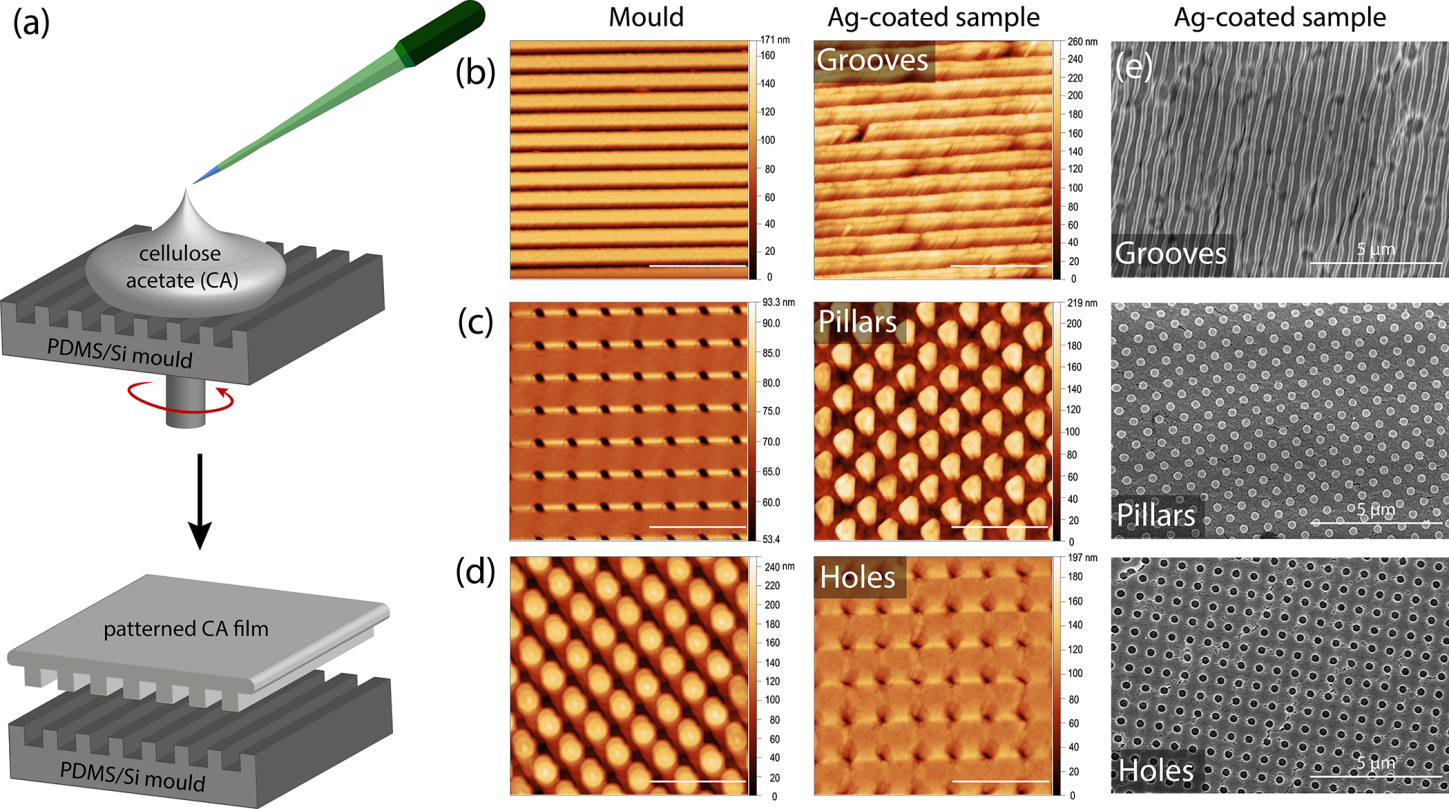
(a) Schematic illustrating the replica molding procedure used for the fabrication of patterned CA films. AFM images of the molds and the samples coated with 10 nm of silver with (b) groove, (c) pillar, and (d) hole-type features (the scale bar represents 2 μm). (e) SEM images of the Ag-coated groove, pillar, and hole CA samples.

Figure 1a shows a schematic illustrating the fabrication process in which either the PDMS stamp or a patterned silicon substrate is used as a mold for the fabrication of CA samples. CA solution in acetone was poured directly on top of the molds and spin-coated until the solvent was completely evaporated. Subsequently, the samples were peeled from the substrate and coated with a 10 nm layer of silver. Figure 1b–d shows atomic force microscopy (AFM) images of the silver-coated CA samples with groove, pillar, and hole-like features, respectively, as well as the molds that were used to prepare them. The images confirm that the arrays of features in the stamp were replicated uniformly with high precision. The features in the sample are preserved after coating the samples with a thin layer of silver, although the voids are filled with the material, resulting in a slight change in the periodicity values for the different substrate designs. The height AFM images were used to determine the size and periodicity of the features; the pillar sample was characterized by the periodicity of 660 ± 40 nm with the features being 127 ± 9 nm high and 420 ± 20 nm wide. The holes in the second sample were characterized by a mean height of 80 ± 10 nm, a width of 400 ± 40 nm, and a periodicity of 760 ± 20 nm. The sample with grooves had the lowest periodicity of 390 ± 10 nm with the features being 70 ± 10 nm high. Additional AFM images of molds and coated and uncoated samples obtained for different scan sizes, demonstrating the high uniformity of the features, as well as line profiles used for size determination are shown in Figures S2–S6. Figures 1e and S7–8 show scanning electron microscopy (SEM) images of the fabricated substrates. Individual defects in the structure of the fabricated nanofeatures, such as cracks and impurities, most likely associated with damage done while the sample is peeled from the mold are visible. The substrates are however highly uniform, which is particularly relevant for the large fabrication area of around 1 cm × 1 cm being imprinted, which illustrates the advantage of the simple replica molding fabrication method.

**Figure 2:**
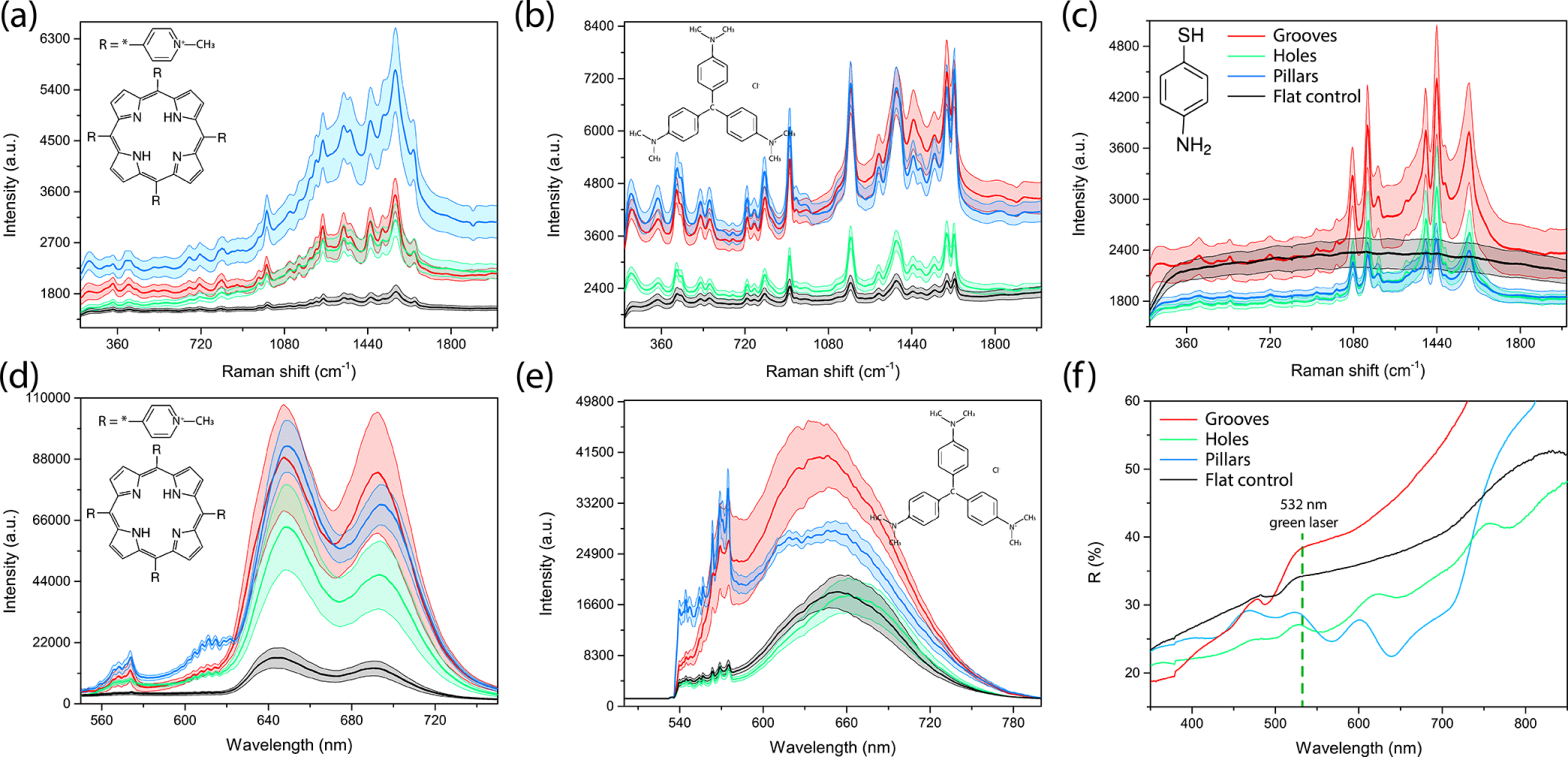
(a) SERS spectra of 10^–4^ M (a) TMPyP, (b) CV, and (c) 4-ABT on Ag-coated CA samples imprinted with different patterns compared to a flat control sample. Fluorescence spectra of (d) TMPyP and (e) CV on the same substrates. (f) Relative reflectance spectra of the substrates collected using a mirror background as a reference.

Following fabrication, we determined the efficiency of the Ag-coated CA samples with different features as substrates for Raman and fluorescence signal enhancement. Three different molecules meso-tetra(*N*-methyl-4-pyridyl)porphine tetrachloride (TMPyP), crystal violet (CV), and 4-aminobenzenethiol (4-ABT) at a concentration of 10^–4^ M were drop-cast on the substrates. Figure 2a–c shows the SERS spectra of the analytes on three different imprinted substrates compared to a flat CA sample spin-coated on a glass substrate coated with silver. The Raman spectrum of TMPyP is characterized by C-pyrrole bending at 1246 cm^–1^ and C–C stretching at 1452 and 1559 cm^–1^, as well as pyrrole bending at 1637 cm^–1^, in agreement with the values reported in the literature.^47^ CV is characterized by peaks at 428 (C–N bending), 807 cm^–1^ (C–H bending), 916 cm^–1^ (radical-ring skeletal vibration), and 1177 cm^–1^ (C–H bending), as well as 1376, 1591, and 1626 cm^–1^ ascribed to C–C stretching vibration.^48^ Both the overall intensity of the peaks of the spectrum and the background-subtracted relative peak intensity increase for the used analytes following deposition on the nanostructure templates. For TMPyP, the relative intensity of the peaks was enhanced almost 8-fold for the pillar sample followed by 5.5-fold enhancement for grooves and 3-fold by holes. Similar relative enhancement values were obtained for CV with the highest enhancement observed for pillars (7-fold).

The SERS spectrum of 4-ABT is characterized by peaks at 1075 and 1579 cm^–1^ assigned to its A_1_ modes.^49^ 4-ABT is known to dimerize into a new compound known as DMAB because of an oxidation process, which takes place following the decay of localized plasmons in metal and generation of hot charges, which can drive chemical reactions.^50^ The additional Raman bands associated with the presence of DMAB located at 1140 cm^–1^ (C–H bending) as well as those at 1392 and 1440 cm^–1^ (stretching vibration of N=N) can be observed in the spectra of 4-ABT on the imprinted Ag-coated samples. In contrast, no peaks are observed for a flat control sample proving that the nanostructuring process is necessary to ensure a plasmon excitation and that the plasmons are generated for all the imprinted samples.

The enhancement in the fluorescence signal was also investigated, as shown in Figure 2d,e. The fluorescence spectrum of TMPyP consists of two broad peaks assigned to Q(0,0) and Q(0,1) bands, centered around 643 and 692 nm, respectively.^51^ The emission peak of CV is centered around 640 nm, in agreement with the values reported in the literature.^52^ The data suggest that the fluorescence signal is increased similarly to the Raman signal, with the highest enhancement being observed for pillars and grooves (5.5-fold) followed by holes (4-fold) for TMPyP as an analyte molecule. The fluorescence of CV was enhanced 2-fold by grooves and 1.5-fold for pillars with the signal strength remaining almost the same for holes. The signal boosting of the Raman and fluorescence signals can be attributed to the same enhancement mechanism, which is the excitation of strong electromagnetic fields close to the surface of the patterned substrates because of the excitation of plasmons in the metal, which enhances the strength of optical signals generated.

Figure 2f shows the specular reflection spectra obtained using a mirror background reference, collected in the visible light range for the different Ag-coated substrates. Dips in the reflection spectrum indicate efficient coupling of the incident light and excitation of an LSPR.^9^ The spectrum shows multiple peaks and valleys in the reflection of Ag-coated samples with grooves and pillars, suggesting the generation of multiple plasmon modes. The dips are located at 488 nm for the substrate with grooves and 552, 646, and 780 nm for holes and for 501, 566, and 638 nm for pillars, with no strong characteristic features being observed for the flat control samples. The features in the reflectance spectra generated for the three designs are homogeneous for the entire surface of the substrates, as shown in Figure S9 depicting the relative reflectance spectra collected from 10 randomly selected spots in the sample. The green laser light (532 nm) used for excitation for Raman and fluorescence data collection was relatively close to the 566 nm dip in the reflection spectrum of Ag-coated CA pillars, ensuring coupling of the light with plasmon modes, which is consistent with the fact that the optical signals were significantly enhanced for analytes deposited on that sample. Because the substrate with imprinted pillars was characterized by the highest enhancement in Raman and fluorescence spectra (next to the groove sample) along with high uniformity in features following the fabrication process, as shown in the AFM images, the subsequent analysis focused on the use of the Ag-coated pillar CA template.

The thickness of the silver coating used for the experiments was determined based on supporting reflectance, SERS, and SEM data shown in Figures S10 and S11. The SEM images show that when a thicker coating is evaporated on the sample, the pillars become “buried” under a layer of metal, while the distance between the individual features becomes significantly reduced. As a result, the dips in the reflectance spectra become less prominent and their position is shifted. For a 50 nm-thick coating, the dips in the spectrum are observed for 649 and 736 nm, which is far from the green laser light wavelength used in the study. For the thickest 100 nm coating, no features in the reflectance spectra can be observed. Consequently, the SERS spectra of TMPyP on samples with a thicker coating are characterized by a lower intensity, as the light used for excitation does not couple with plasmon modes in the sample. The relatively thin 10 nm silver coating used allows preserving the shape, size, and periodicity of the features of the mold used during the replica molding procedure, while also being thick enough to enable a plasmon excitation in the metal.

**Figure 3:**
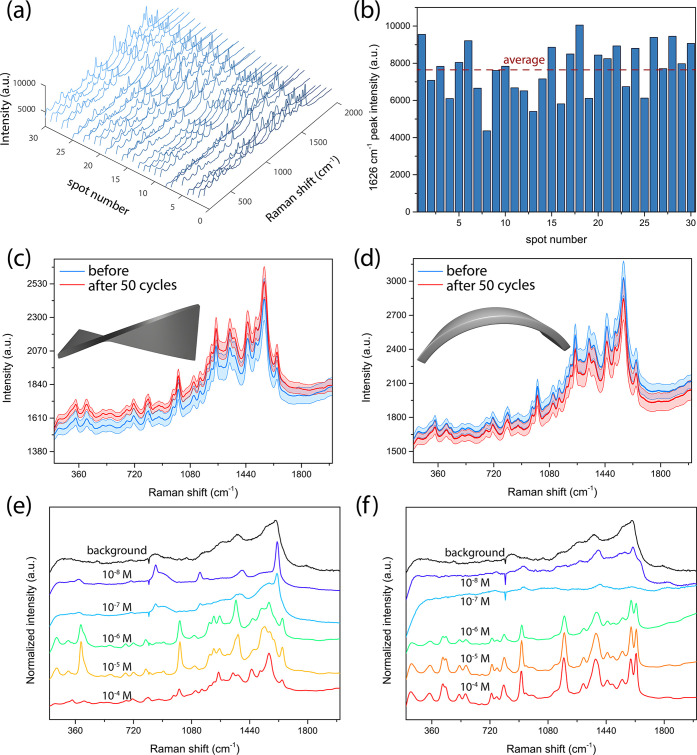
(a) SERS spectra from 30 randomly selected spots on the Ag-coated pillar CA sample with 10^–4^ M CV drop-cast on top. (b) Intensities of the 1626 cm^–1^ peak measured on the different spots of the sample compared to the average signal. The SERS spectrum of 10^–4^ M TMPyP on the Ag-coated pillar CA substrate prior to and after 50 cycles of (c) torsion and (d) bending. Normalized SERS spectra of (e) TMPyP and (f) CV deposited at different concentrations on the Ag-coated pillar CA sample used to determine the detection limit.

Subsequently, we analyzed the reproducibility of the Raman signal collected from analytes deposited on the manufactured substrates. To make SERS substrates viable for field or nonlab qualitative analysis, they should have low variation in the strength of generated signals obtained from different spots on the sample. Additionally, templates resulting in Raman signals with less than 15% relative standard deviation could be used for quantitative analysis.^53^Figure 3a,b shows the spectra from 30 randomly selected spots on the Ag-coated pillar CA sample with CV used as an analyte molecule as well as the intensity of the strongest peak in the spectrum compared to the mean value. Additional data for TMPyP and 4-ABT are shown in Figure S12. A relative standard deviation of 17% for TMPyP, 18% for CV, and 13% for 4-ABT was obtained, reaching the values desired for quantitative Raman studies. The values confirm that the manufactured substrates are highly uniform. The small variations in the signal could be explained by different strengths of electric field being generated in the flat areas of the sample compared to the pillars themselves. It has been previously shown that both areas for a substrate consisting of metallic nanopillars can be plasmon-active because of the generation of different modes, which can be coupled to different wavelengths of light.^20^

The mechanical stability of the substrates was also tested. One advantage of using imprinted polymer-based substrates instead of metal-only substrates prepared using lithography methods is flexibility. The flexibility of the substrates is of high importance, as it allows for performing on-field Raman measurements for chemical and biological molecule detection applications.^12^ More conventional substrates are often prepared on silicon and glass, reducing their applicability. We collected the average signal from TMPyP deposited on the substrate prior to and after 50 cycles of torsion (Figure 3c) and bending (Figure 3d). Following the repeated mechanical deformation, no significant changes in the spectrum were observed, and no significant damage was done to the samples as demonstrated in Figures S13–S15 showing the SEM images and reflectance spectra of the samples following deformation. The CA-based samples could therefore be used to cover the surface of a curved object^54^ and collect molecules for analysis and then be used for SERS-based sensing without any loss in the structural integrity of the substrate.

Finally, we determined the detection limit for TMPyP and CV deposited on the Ag-coated CA pillars. Figure 3e,f shows that both molecules can be detected at the lowest concentration of 10^–6^ M. When the concentration is reduced further, only peaks at 857, 1132, 1396, and 1610 cm^–1^ can be observed, which can be ascribed to the Ag-coated CA substrate, as they are also present when no molecule is deposited on the sample. On top of the relatively low limit of detection, the samples are also characterized by high stability when exposed to high-intensity laser light up to 20 mW, as shown in Figure S17.

**Figure 4:**
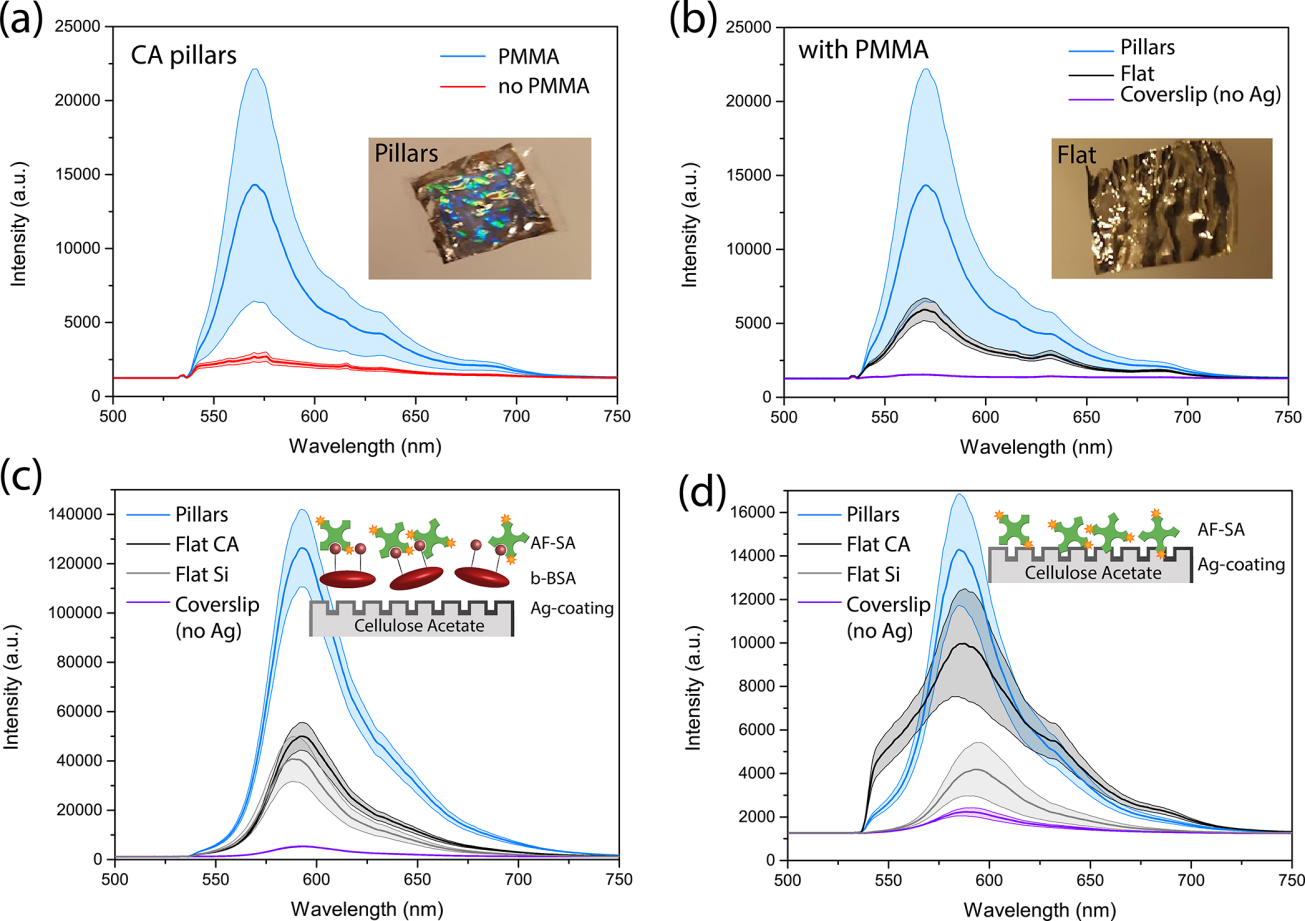
(a) SEF spectra of 10^–7^ M RhB (a) on the Ag-coated pillar CA sample with and without the use of PMMA and (b) with PMMA on different substrates including Ag-coated CA pillars, flat Ag-coated CA, and a coverslip without any metal on it. The insets in (a) and (b) show the images of the Ag-coated pillar CA sample and an Ag-coated flat CA control sample, respectively. SEF spectra of AF-SA deposited on flat and patterned CA-based samples (c) with and (d) without the use of b-BSA.

Although we have previously shown (Figure 2d,e) that the as-fabricated CA-based pillar substrates can be used for fluorescence enhancement following direct deposition of the analytes on the template, we decided to expand on and optimize the approach. To obtain the best signal enhancement for SEF, spacer layers need to be introduced in between the metallic substrate and the probe molecules, to minimize a charge transfer mechanism taking place. Charge being transferred from the analyte to the metal may result in fluorescence quenching because of nonradiative decay; the process can however be regulated by adjusting the distance between the detected molecules and the substrate, reaching values below 10 nm for best enhancement.^55−57^ For that reason, we mixed the analyte molecule with the PMMA polymer prior to deposition, which is known to introduce a nanometer-thick spacing in between the components following spin coating of the analyte molecule solution. Figure 4a shows the SEF spectra of rhodamine B (RhB) spin-coated on the CA-based pillar substrates with and without the use of PMMA. The fluorescence spectrum of RhB is characterized by a broad peak centered around 570 nm, which is consistent with values reported in the literature.^58^ Upon the addition of PMMA to the sample, the signal intensity is enhanced 5-fold, and a clear fluorescence peak associated with the analyte molecule can be seen, which would allow for low-concentration probe molecule detection. Similarly to what we have shown previously in Figure 2, the substrate patterned with nanosized pillars performs significantly better, with 2.4-fold enhancement observed for RhB/PMMA on the silver-coated patterned sample compared to the flat silver-coated CA template. The signal intensity is also 9 times higher than the signal of RhB/PMMA spin-coated on a bare coverslip, without the use of a metal coating.

One application of fluorescence-based sensing of molecules that has become particularly relevant in previous years is the development of immunoassays, which are applicable in many medical fields ranging from biomedical research to diagnostics.^59^ Fluorescence assays are based on the detection of a substance using the selective binding of a given protein to an antibody or an antigen. Two approaches can be used for the design of a fluorescence immunoassay; either the investigated molecule is directly labeled with a fluorescent dye and immobilized on a substrate, or a sandwich structure is used, in which the presence of a molecule is detected by collecting the signal from secondary fluorescent antibodies bound to it.^60^ Metal-based patterned substrates have previously been used as supporting substrates for such assays, which can be used to immobilize molecules on the substrate and enhance the fluorescence signal intensity, enabling the detection of molecules.^61^ The traditionally used SEF platforms are however limited in their use as immunoassay templates, by the methods necessary for their fabrications.^59^

We further expand on our approach of employing the Ag-coated imprinted cellulose-based samples for fluorescence detection, and we show that the templates can be used for immobilization and detection of Alexa Fluor-conjugated streptavidin. Streptavidin is a protein often used in the development of model immunoassays, because of its high affinity to biotin, also known as vitamin B_7_, which is an important biomolecule, responsible for various metabolic processes. The interaction between streptavidin and biotin is a protein–ligand interaction, which has been previously used for the detection of proteins, antibodies, and enzymes, and is one of the strongest noncovalent interactions known in nature.^62^ Streptavidin conjugated with a fluorescent dye has been previously immobilized and detected on metal-based SEF platforms.^63,64^ In the specific approach, biotinamidocaproyl-labeled bovine serum albumin (biotinylated-BSA; b-BSA) is often used, because of its affinity to bind to metal complexes such as metal and gold as well as glass.^65^ Deposition of b-BSA on a metal substrate facilitates the fabrication of a monolayer that can scavenge streptavidin- or avidin-conjugated markers from solution and immobilize them on the substrate, enabling their detection using fluorescence methods. Dyes such as Alexa Fluor protein conjugates have previously been detected on Ag metallic surfaces fabricated using lithography methods, resulting in an average 8 to 80-fold enhancement of the signal, depending on the size and the shape of the manufactured nanofeatures.^64^

Figure 4c shows the fluorescence spectrum of the model immunoassay consisting of an Alexa Fluor 568 streptavidin-conjugated (AF-SA) dye deposited on b-BSA with the supporting Ag-coated pillar CA substrate compared to the fluorescence signal from nonpatterned control samples. Both a flat CA sample spin-coated on glass and a piece of a flat silicon wafer coated with silver were used as control samples, resulting in similar fluorescence signal strength, indicating that the material used underneath the coating does not affect the enhancement significantly. When no metal coating was used, however the signal intensity was significantly lower, because of the reduced binding of b-BSA to the supporting substrate and therefore the lack of binding sites for the fluorescent AF-SA. The imprinted CA sample with pillars was characterized by the best performance with the signal strength being enhanced 3-fold when compared to the flat samples with metal and 23-fold relative to the flat sample without metal. Figure 4d shows the fluorescence spectra of AF-SA deposited directly on the imprinted CA and control samples without the use of b-BSA. The spectra show a similar strength signal compared to the samples with b-BSA, suggesting that AF-SA can become immobilized on the imprinted substrate even without the use of biotin, and it is not removed during washing of the sample. We can infer that the imprinting of the sample with nanofeatures not only results in signal enhancement but also acts as a rough substrate with many crevices that can confine the molecules deposited on the platform, limiting their removal.

**Figure 5:**
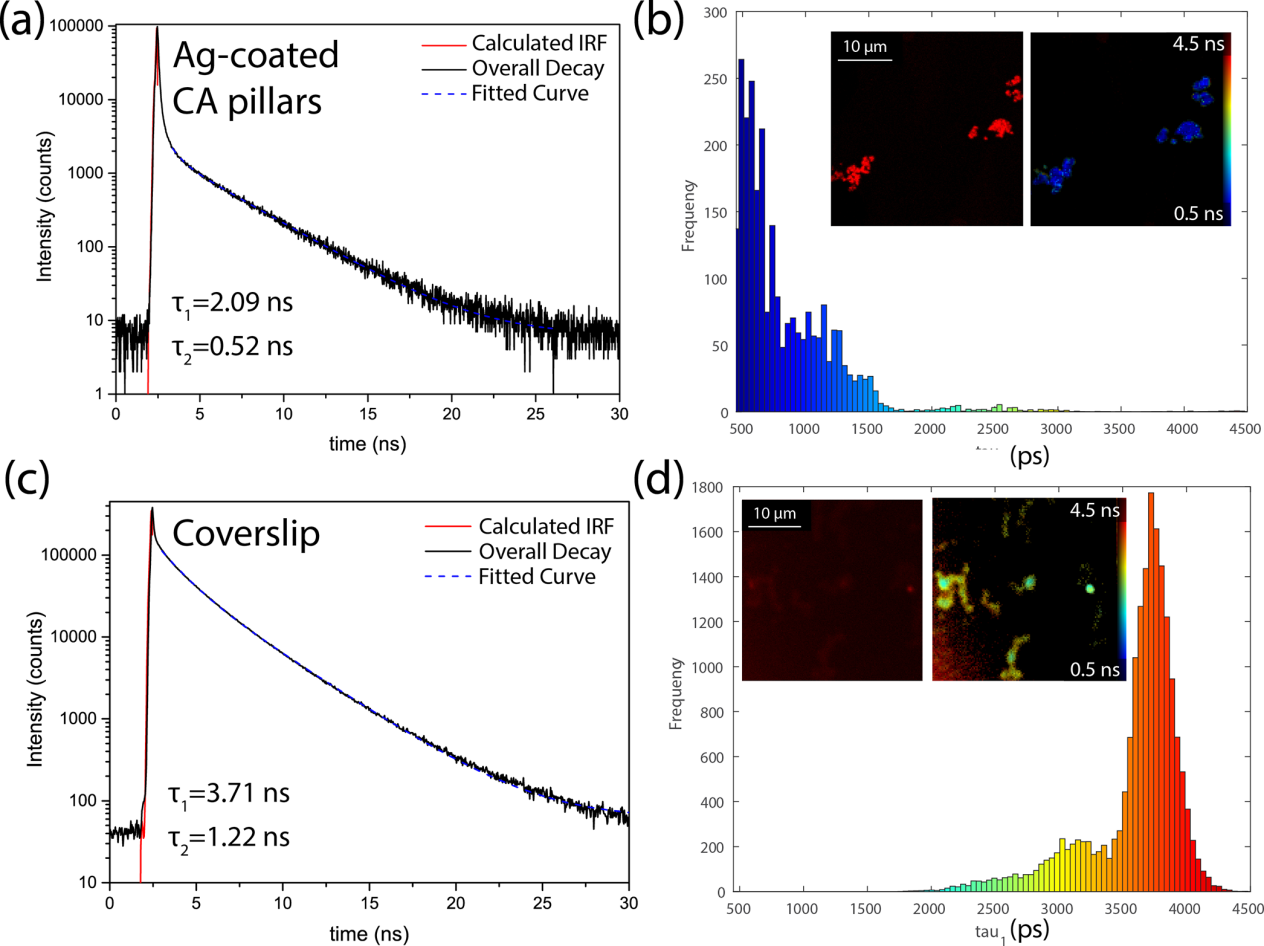
(a, d) Fluorescence decay curves along with the lifetimes calculated, and (b, d) lifetime histograms of the AF/b-BSA assays on (a, b) Ag-coated CA-pillar sample compared to a coverslip without any metal on it (c, d). The insets in (b) and (d) show the confocal and fluorescence lifetime images collected for the samples; 568 nm excitation wavelength was used for all the measurements.

When a fluorescent system such as the dye used in the study absorbs a photon, it spontaneously emits a photon of lower energy after an average time known as the lifetime. The time the molecule stays in the excited state depends on the local environment of the fluorophore and therefore provides additional information on the interaction between the dye and the substrate it is deposited on. To better understand the origin of the fluorescence enhancement observed on the imprinted CA samples, we determined the fluorescence lifetimes for the AF/b-BSA assays prepared on the Ag-coated CA pillars compared to the assay prepared on a metal-free coverslip. The data collected using fluorescence lifetime imaging (FLIM) by the multidimensional time-correlated single-photon counting technique for the two samples investigated are shown in Figure 5. According to the manufacturer, the fluorescent AF-SA is characterized by a lifetime of 3.6 ns, which is consistent with the values of τ_1_ = 3.71 ns and τ_2_ = 1.22 ns we determined from the fitting of the fluorescence decay curve for AF-SA on b-BSA on a coverslip. The lifetime of the dye decreases significantly and reaches the values of τ_1_ = 2.09 ns and τ_2_ = 0.52 ns when the imprinted silver-coated CA substrate is used. Reduction of a fluorescence lifetime is associated with increased fluorescence signal intensity, and it was previously widely reported for molecules in the presence of plasmonic nanostructures.^66^ The decrease of the lifetime increases the radiative decay rates as it allows the fluorophore to undergo more excitation–de-excitation cycles, which increases the total number of photons emitted by the sample, resulting in higher brightness. Additionally, a shorter lifetime increases the photostability of a dye because photochemical degradation takes place when the molecule is in the excited state.^56^ Reducing the time the fluorophore stays in the excited state delays the photobleaching process, which is a permanent process reducing fluorescence intensity because of photon-induced breaking of covalent bonds and chemical damage.^67^ Based on the reduction of lifetime values of a dye in the proximity of the silver-coated CA pillars, we can infer that the described process is responsible for the observed fluorescence enhancement for the AF-SA assays investigated.

## Conclusions

We have demonstrated that large-surface area plasmonic crystals can be fabricated by metal coating nanopatterned CA substrates, prepared using soft nanoimprint lithography methods. The substrates can be patterned into various uniform and regular shapes including grooves, pillars, and holes with the period of the features in the 400–700 nm range by simply spin coating the polymer on a mold. The described method is a cost-effective alternative to traditional optical lithography for the fabrication of plasmonic templates, which also benefits from the nontoxicity, biodegradability, biocompatibility, and renewability of the cellulose-based polymer used. We have shown that the fabricated templates are plasmon-active and can be used as substrates for Raman- and fluorescence-based molecule detection. Because the substrates do not dissolve in water, various molecules can be directly deposited in solution on the fabricated templates and detected at concentrations as low as 10^–6^ M via Raman spectroscopy and 10^–7^ M via fluorescence spectroscopy. The substrates are flexible and mechanically stable under repeated cycles of bending and torsion and provide a highly reproducible signal that can be used for low-concentration molecule detection in fields such as environmental monitoring of water pollutants and medical diagnostics. We have shown that the fluorescent signal of molecules can be enhanced by introducing a spacer PMMA layer that reduces fluorescence quenching because of nonradiative decay. Additionally, the fabricated Ag-coated CA substrates patterned with nanopillars can be used as supporting substrates for immunoassays utilizing the streptavidin–biotin interaction for AF-SA detection with signal enhancement as high as and 23-fold relative to a flat sample without metal. The model assay shows the potential of the platform to be further developed as a sensing platform for the detection of antibodies, enzymes, and other important biomolecules and therefore can be used in the medical field for the design of assay-based diagnostic tools.

## Experimental Section

### Stamp Preparation

II-VI Aerospace & Defense provided a 2D silicon nanostamp with Rect Post features (S2D-24B3-0808-150-P; 150 nm groove depts, 260 nm post width, 700 nm period) and a linear silicon nanostamp (SNS-C24-1212-110-P; 110 nm depth, 417 nm period). Composite PMDS stamps consisting of a thin hard PDMS (h-PDMS) layer to ensure a uniform replica of the pattern as well as a thicker soft PDMS (s-PDMS) to allow removal of the stamp without breakage were fabricated using a method reported elsewhere.^43^ High-modulus reprographic silicone (hPDMS; product code: PP2-RG07) was provided by Gelest while soft PDMS (sylgard 184; product code: 761036) was acquired from Sigma-Aldrich.

### Replica Molding of CA Films

The fabrication process of nanoimprinted cellulose derivative samples is described in detail elsewhere.^20^ Cellulose acetate powder (CA; average *M*_n_ ∼ 30,000 obtained by gel permeation chromatography; CAS Number: 9004-35-7) was provided by Sigma-Aldrich. A solution of the polymer was prepared by mixing 0.64 g of the CA powder with 5.333 mL of acetone, which was then stirred overnight. The solution was then poured either directly on the silicon nanostamp or on the fabricated PDMS mold and spin-coated at 2 V for about 1 min. The samples were then peeled from the stamp. A bespoke silver evaporation system with a Sycon Instruments STM-100/MF thickness monitor was used to coat the samples with 10 nm of silver.

### Preparation of Probe Molecule Solutions

Meso-Tetra (*N*-methyl-4-pyridyl) porphine tetrachloride (TMPyP; T40125, Frontier Scientific) and RhB (CAS: 81-88-9; Sigma-Aldrich) solution were prepared by dissolving the powder in distilled water at an initial concentration of 10^–2^ M. 4-ABT (CAS 1193-02-8; Sigma-Aldrich) was dissolved in methanol at an initial concentration of 10^–2^ M. CV 1% aqueous solution (CAS Number: 548-62-9) was also used. All the analyte molecule solutions used were diluted down to the desired concentration using distilled water and then drop-cast on the previously prepared substrates. PMMA (CAS: 9011-14-7) was dissolved in toluene (Fisher Scientific, laboratory reagent grade, CAS: 108-88-3) at a concentration of 5 wt/vol %. For fluorescence measurements, 10^–7^ M RhB was mixed with the PMMA solution or with distilled water prior to being spin-coated on a substrate.

### Preparation of Assays

b-BSA (Thermo Scientific Pierce 29130) and streptavidin-conjugated dye, Alexa Fluor 568 conjugate (AF-SA; Molecular Probes S11226) were provided by Fisher Scientific; 20 μL of 2 mg/mL b-BSA solution in distilled water was drop-cast on the Ag-coated CA surface and incubated for 1 h and subsequently rinsed with deionized water to remove unbound proteins. Twenty microliters of 0.1 mg/mL AF-SA solution in phosphate-buffered saline (PBS; Alfa Aesar J62692, 10X, pH 7.6) was then added to the surface, incubated for 2 h, and then rinsed with water to remove any unbound molecules.

### Raman and Fluorescence Spectroscopy

The measurements were performed using a bespoke Raman system consisting of a monochromatic laser (HeNe, ThorLabs) with a beam splitter and a long-pass filter (RazorEdge, Semrock), an inverted optical microscope (IX71, Olympus), a spectrograph (SP-2300i, Princeton Instruments), and a CCD camera (iDus 401, Andor).^55,68−70^ A 50× objective was used to focus the laser (532 nm wavelength, 5 mW incident power regulated by an attenuator) and collect the Raman and fluorescence signals with an exposure time of 2 s in an accumulation mode (10 accumulations). The CCD camera was calibrated over the spectral window using the Raman spectrum of toluene. To take spatial variability into consideration, an average signal from 10 different spots on the sample was reported.

### Atomic Force Microscopy

AFM images were obtained using an MFP-3D Asylum Research instrument operating in tapping mode. Monolithic silicon Tap300Al-G probes with aluminum reflective coating (BudgetSensors) were used to obtain the images. The tips used were characterized by the following specifications: 40 N/m (20–75 N/m) force constant, 300 kHz (200–400 kHz) resonance frequency, and 125 μm (115–135 μm) length.

### UV–Vis Reflection Spectroscopy

A LAMBDA 750 UV/vis/NIR spectrophotometer (PerkinElmer) was used to determine the specular reflectance. Reflectance spectra from the substrates were measured as a function of Δ*R*/*R* = (*R*_sample_ – *R*_background_)/*R*_background_ using an aluminum mirror as a reference. The spectra were obtained in the 180–1000 nm range for 1 nm step size.

### Fluorescence Imaging

Fluorescence confocal and fluorescence lifetime microscopy images were obtained using the Leica TCS SP8 confocal system using a white light laser set to 568 nm and an internal HyD GaAsP SMD detector. A 100× oil objective was used, and the samples were imaged through a coverslip in air, without the introduction of a mounting medium.

## Supporting Information Available

Figures S1–S17 - Additional experimental data including high-resolution SEM images, AFM images, and associated line profiles, additional reflectance data, as well as SERS data used for the determination of the detection limit, and assessment of substrate stability and reproducibility (PDF)
