# Supplementary material for: Cellulose Acetate-Based Plasmonic Crystals for Surface-Enhanced Raman and Fluorescence Spectroscopy
Source: ACS Mater Au. 2022 Mar 23;2(4):453–63. doi: 10.1021/acsmaterialsau.2c00013 (PMC9928397; doi:10.1021/acsmaterialsau.2c00013)
Supplement: Supplementary file 1 — mg2c00013_si_001.pdf [file mg2c00013_si_001.pdf]

Supporting Information for

Cellulose acetate-based plasmonic crystals for surface enhanced Raman and fluorescence spectroscopy

Agata Fularz,[1], Dimitrios Stogiannis[1,2], James H. Rice*,[1]*

[1] School of Physics, University College Dublin, Belfield, Dublin 4, Ireland.

[2] Department of Physics, University of Ioannina, Ioannina, Greece

*Corresponding Author. E-mail: james.rice@ucd.ie, agata.fularz@ucdconnect.ie

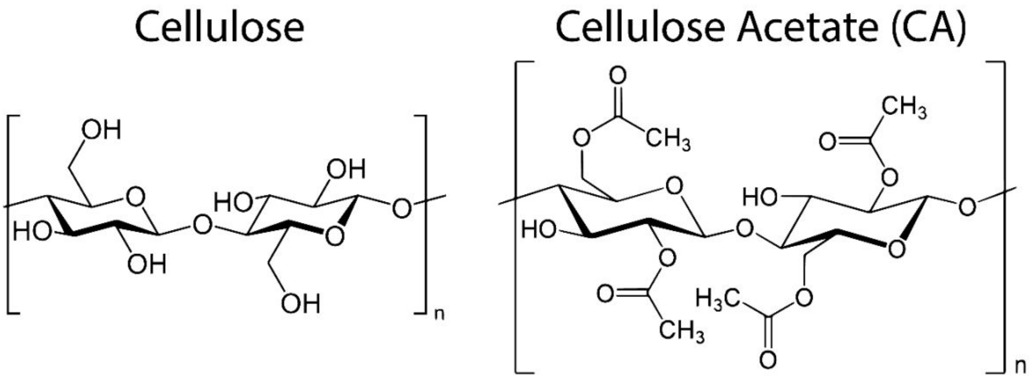

Figure S1. Chemical structure of cellulose and its derivative – cellulose acetate used for the fabrication of nanoimprined films.

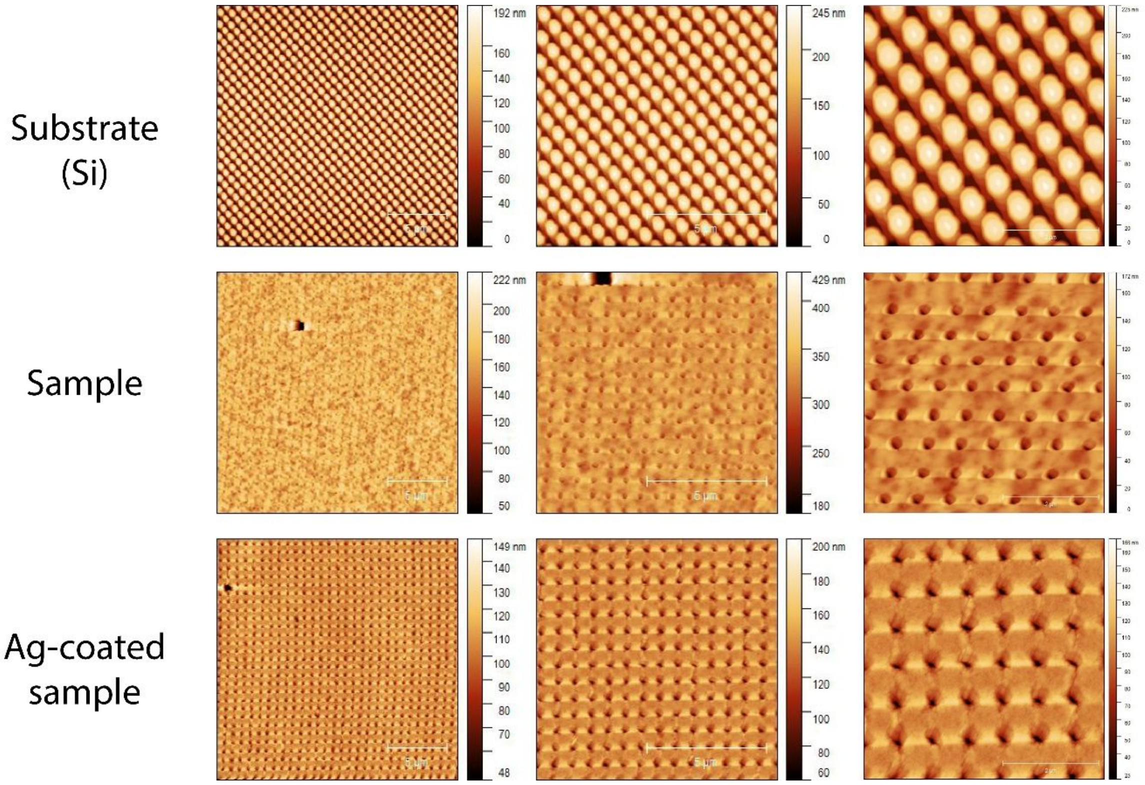

Figure S2. AFM images of the Rect Post silicon nanostamp substrate used for replica moulding as well as the cellulose acetate sample uncoated and coated with 10 nm of silver.

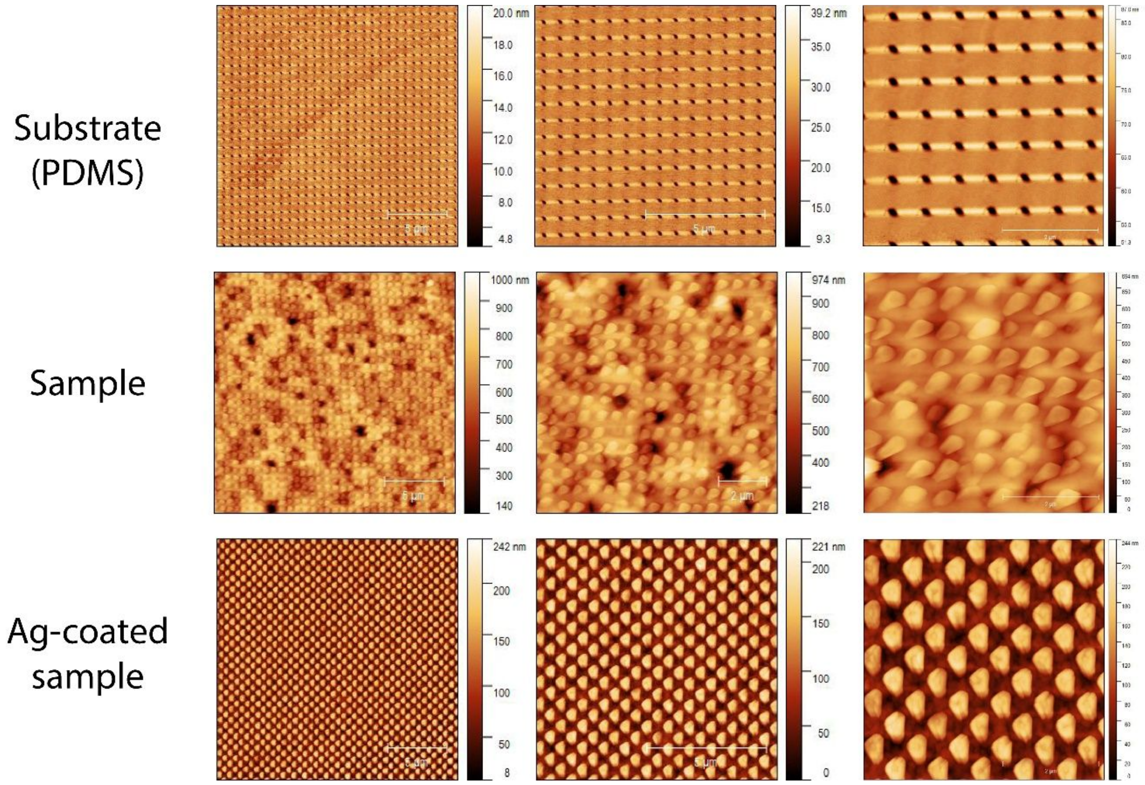

Figure S3. AFM images of the PDMS substrates prepared using the Rect Post silicon nanostamp used for replica moulding as well as the cellulose acetate sample uncoated and coated with 10 nm of silver.

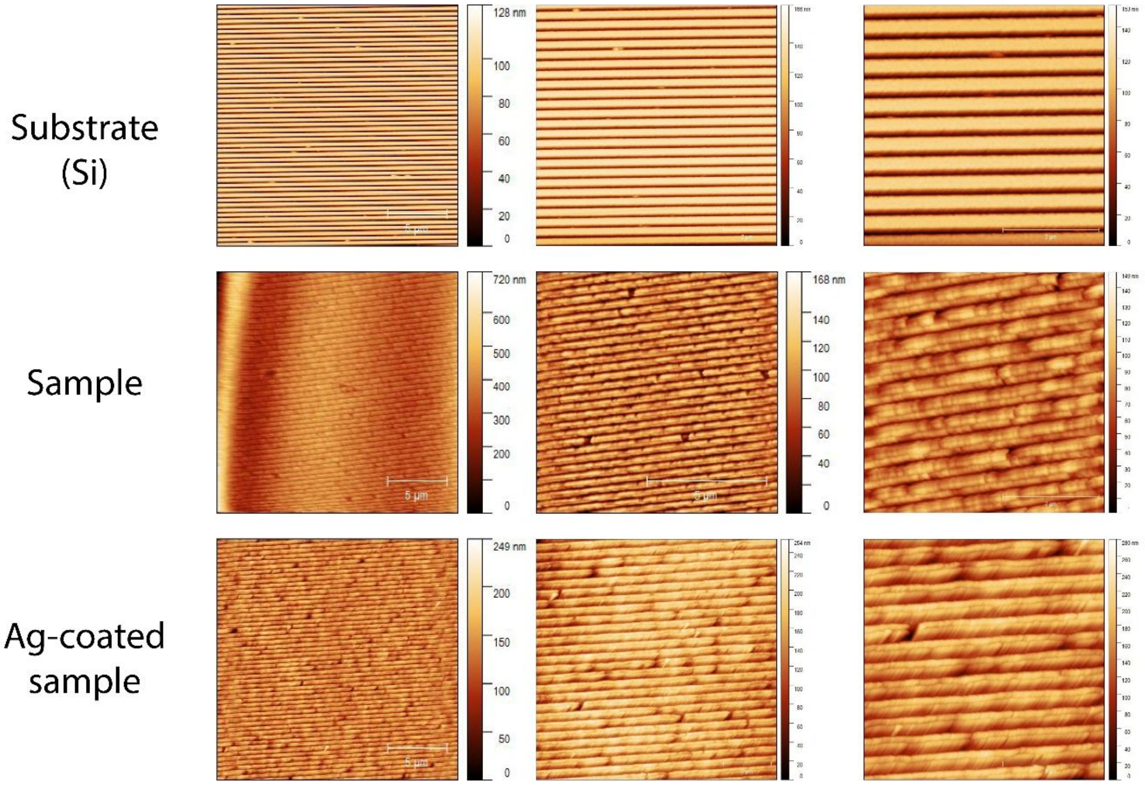

Figure S4. AFM images of the Linear silicon nanostamp substrate used for replica moulding as well as the cellulose acetate sample uncoated and coated with 10 nm of silver.

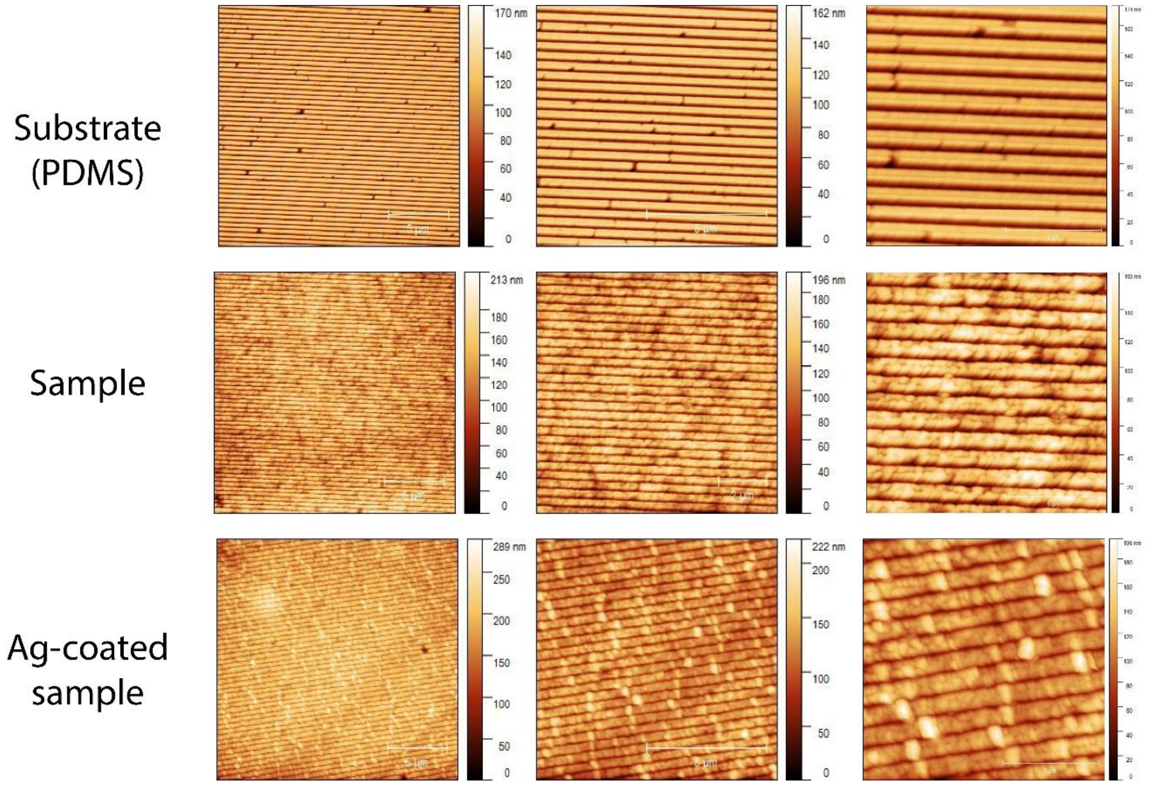

Figure S5. AFM images of the PDMS substrates prepared using the Linear silicon nanostamp used for replica moulding as well as the cellulose acetate sample uncoated and coated with 10 nm of silver.

No significant differences in topography were observed in between the groove CA sample prepared using Si and PDMS moulds, therefore only the samples prepared on the PDMS stamps were further analyzed.

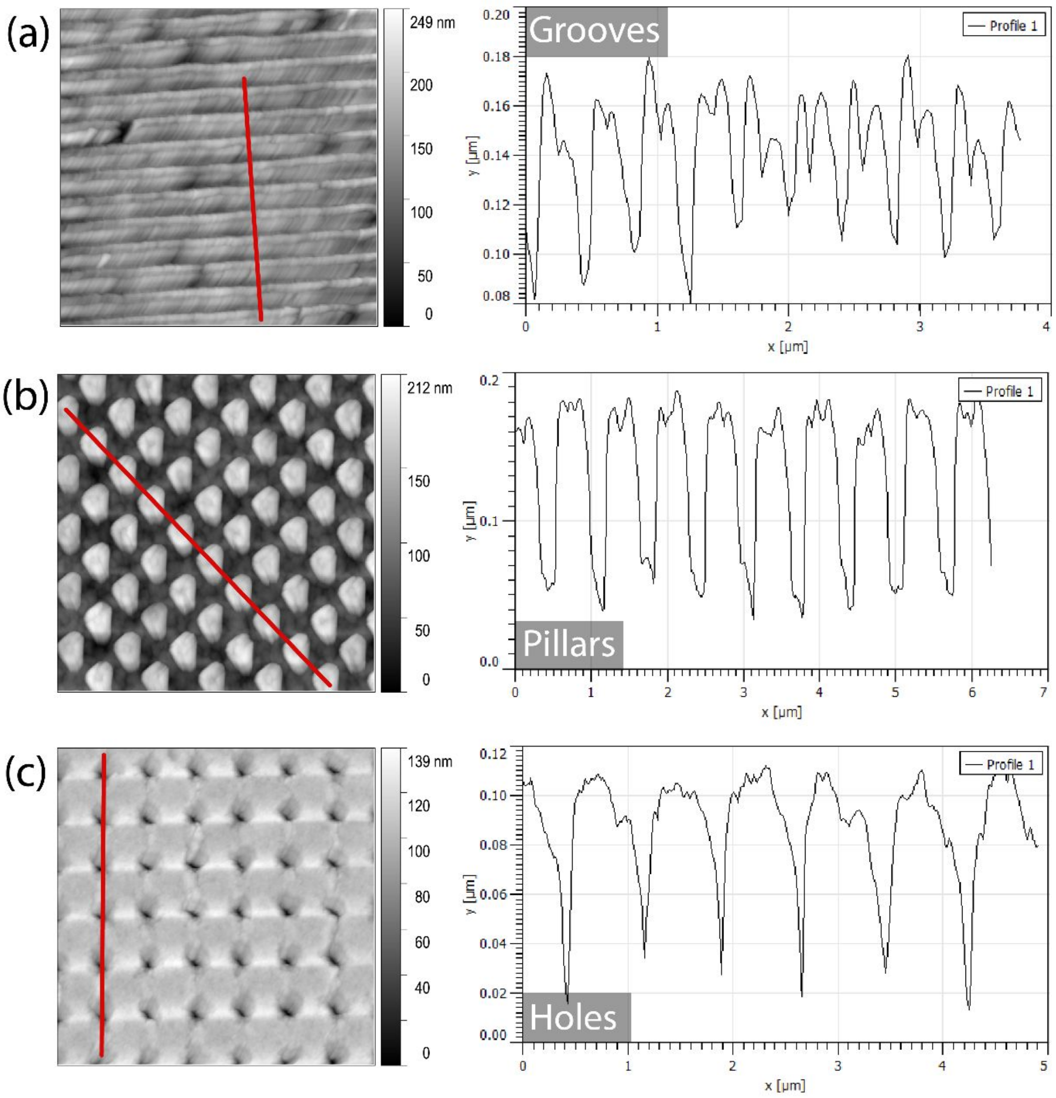

Figure S6. AFM images of the Ag-coated CA samples with (a) grooves, (b) pillars, and (c) holes as well as line profiles used to determine the lattice periods and parameters associated with the features on the samples.

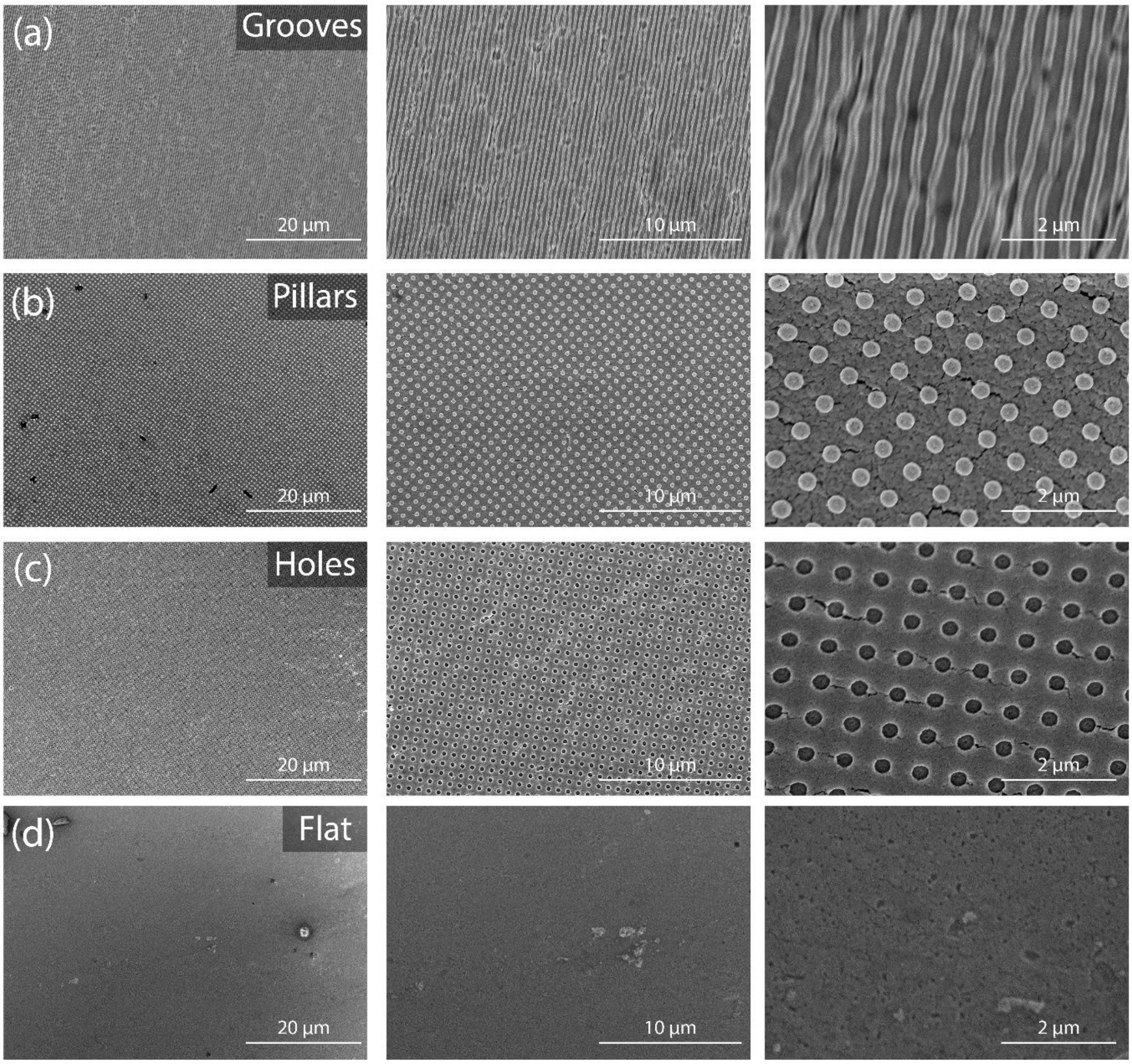

Figure S7. SEM images of the Ag-coated CA samples with (a) grooves, (b) pillars, (c) holes, and (d) flat control samples obtained at different magnifications, showing the high uniformity and large nanopatterning area of the substrate.

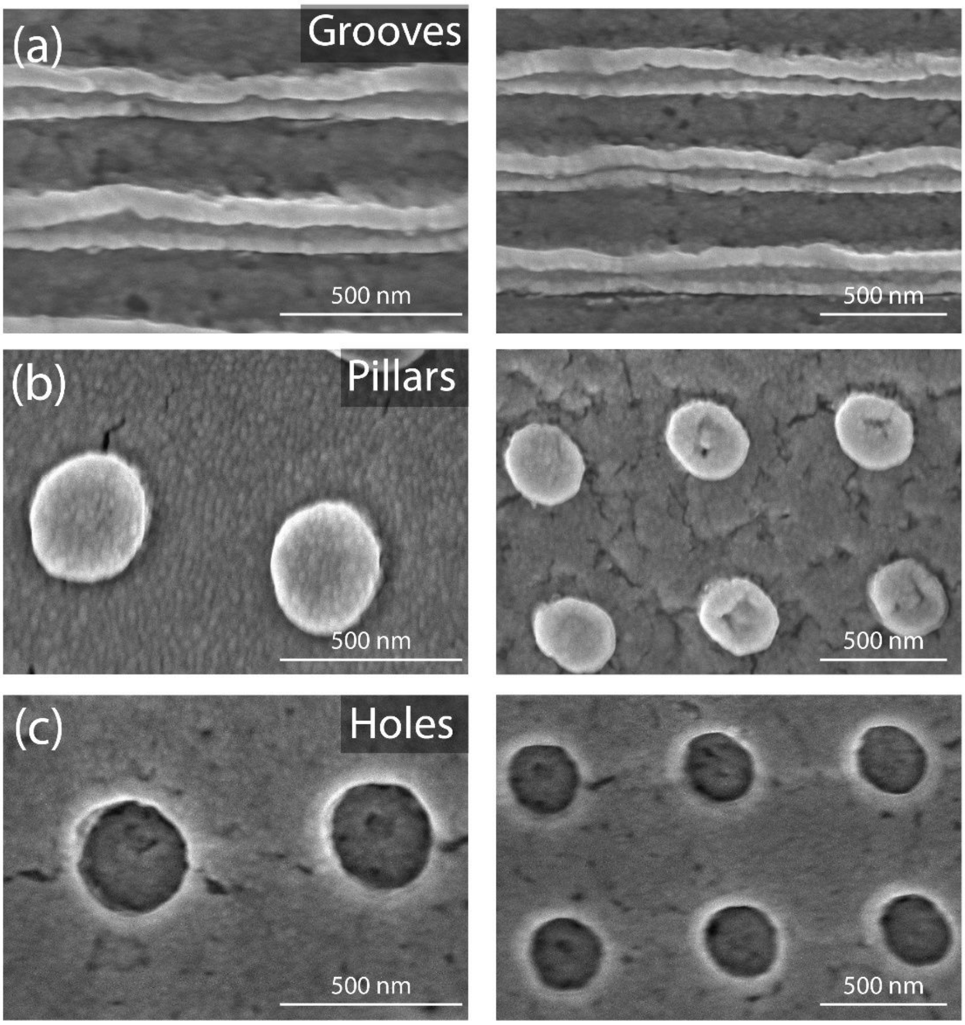

Figure S8. High-resolution SEM images of the Ag-coated CA samples with (a) grooves, (b) pillars, and (c) holes.

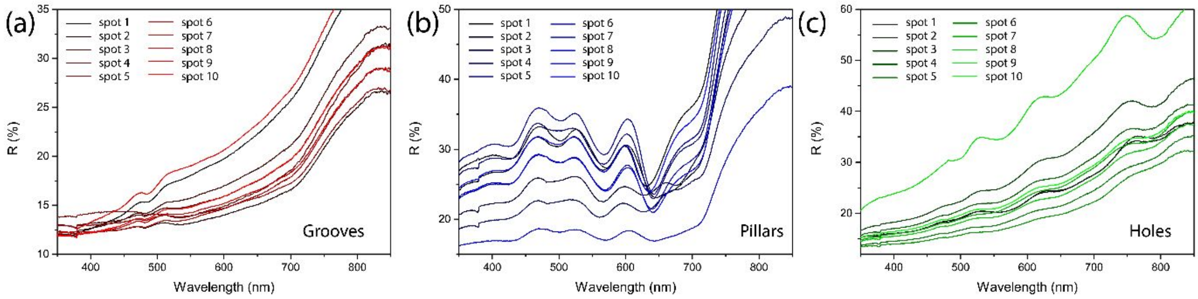

Figure S9. Relative reflectance spectra of the Ag-coated cellulose acetate substrates with (a) groove, (b) pillar, and (c) hole-type features collected from 10 randomly selected spots on the sample.

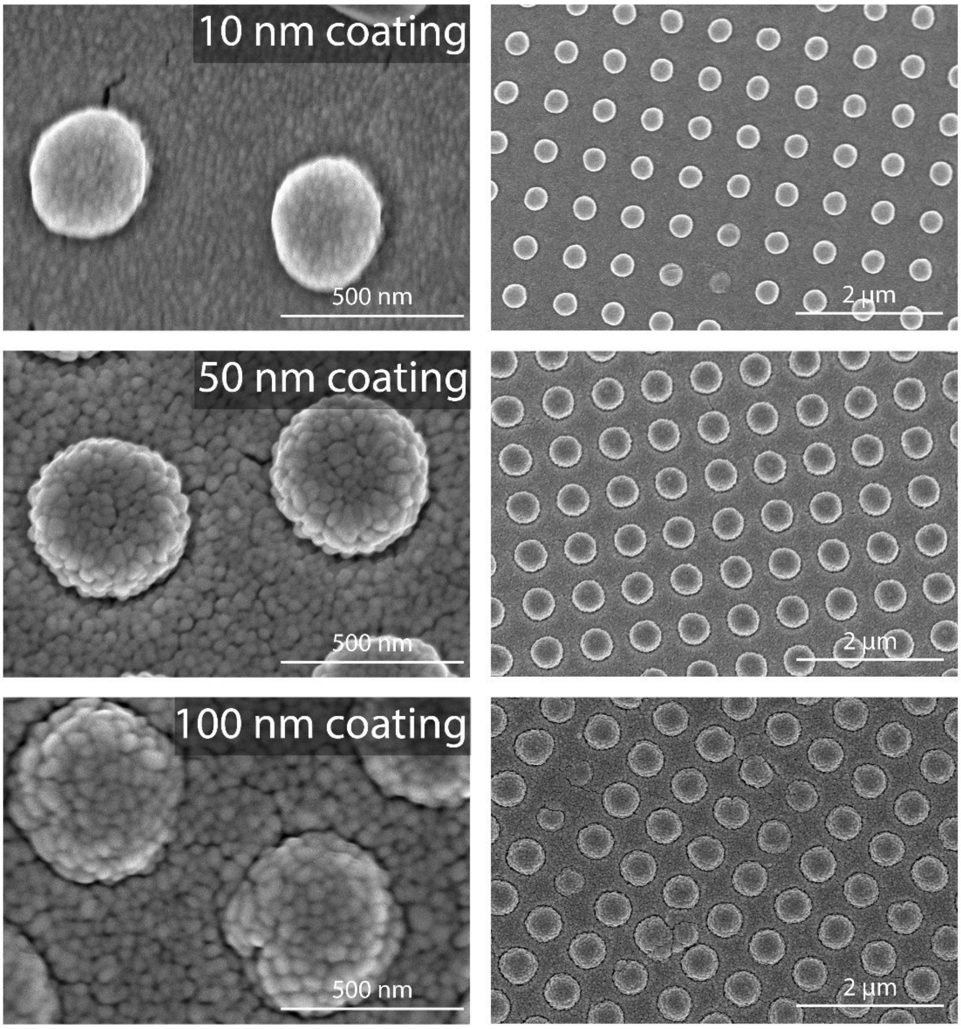

Figure S10. SEM images of the Ag-coated pillar CA sample with a varying thickness of silver coating evaporated on top.

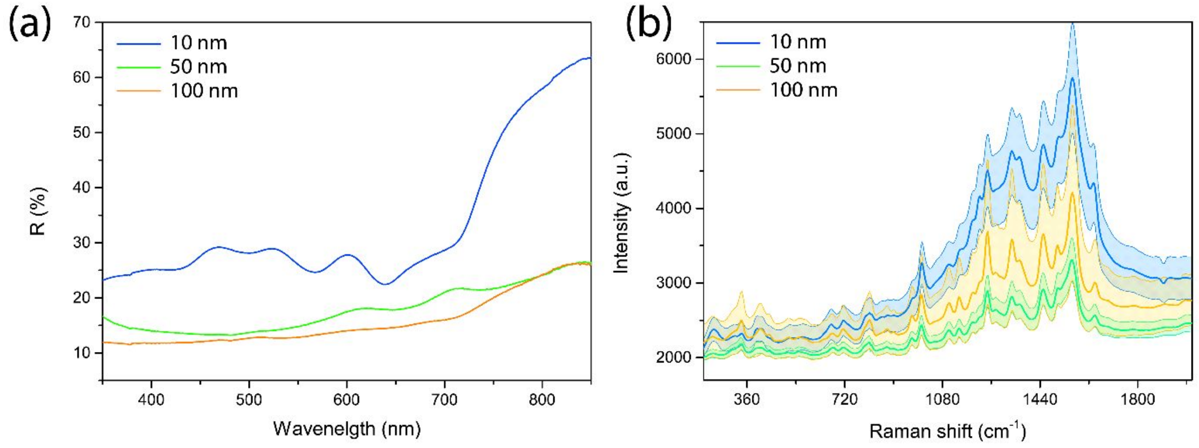

Figure S11. (a) Relative reflectance spectra of the Ag-coated cellulose acetate substrates a varying thickness of silver coating evaporated on top. (b) shows the SERS spectra of 10^{-4} M TMPyP deposited on the samples.

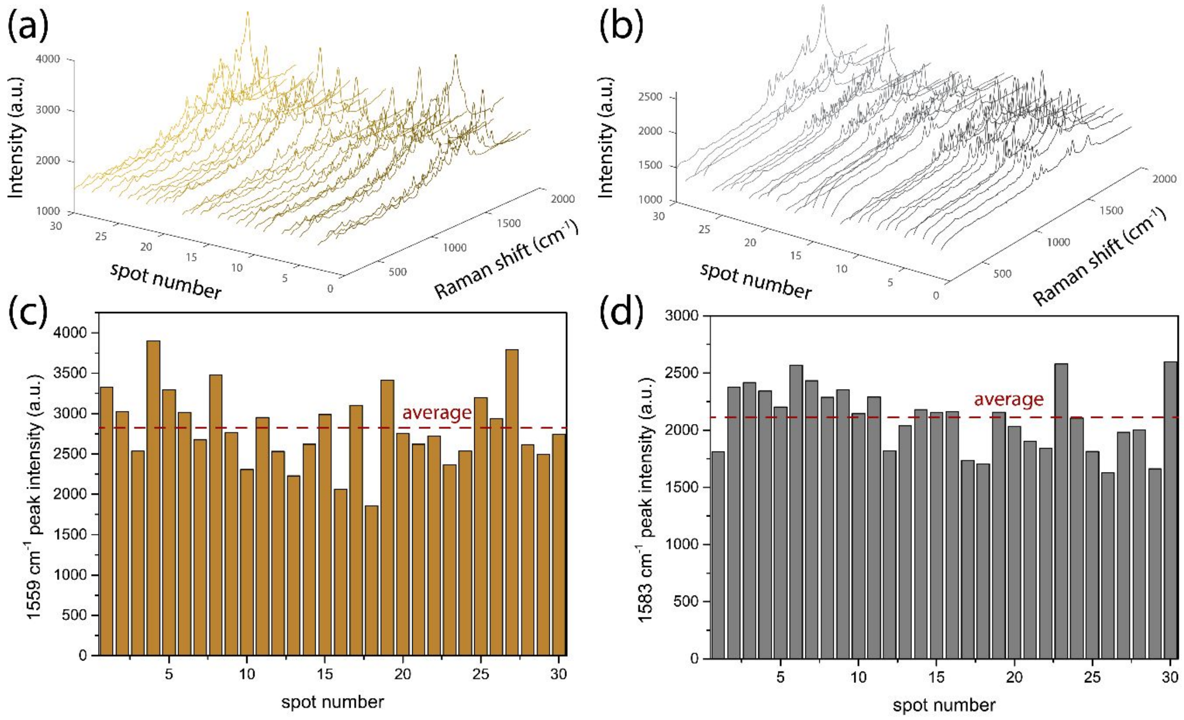

Figure S12. SERS spectra from 30 randomly selected spots on the Ag-coated pillar CA sample with 10^{-4} M (a) TMPyP and (b) 4-ABT drop cast on top. (c) and (d) shows the intensities of the highest intensity peaks measured on the different spots of the sample compared to the average signal for TMPyP and 4-ABT respectively.

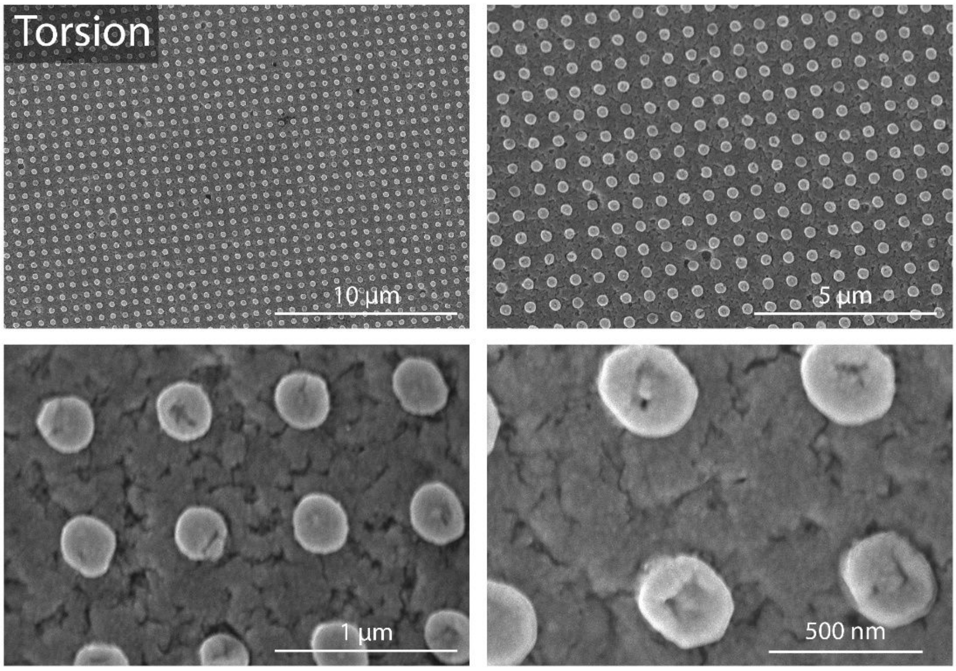

Figure S13. SERS spectra of the Ag-coated pillar CA sample after 50 cycles of torsion.

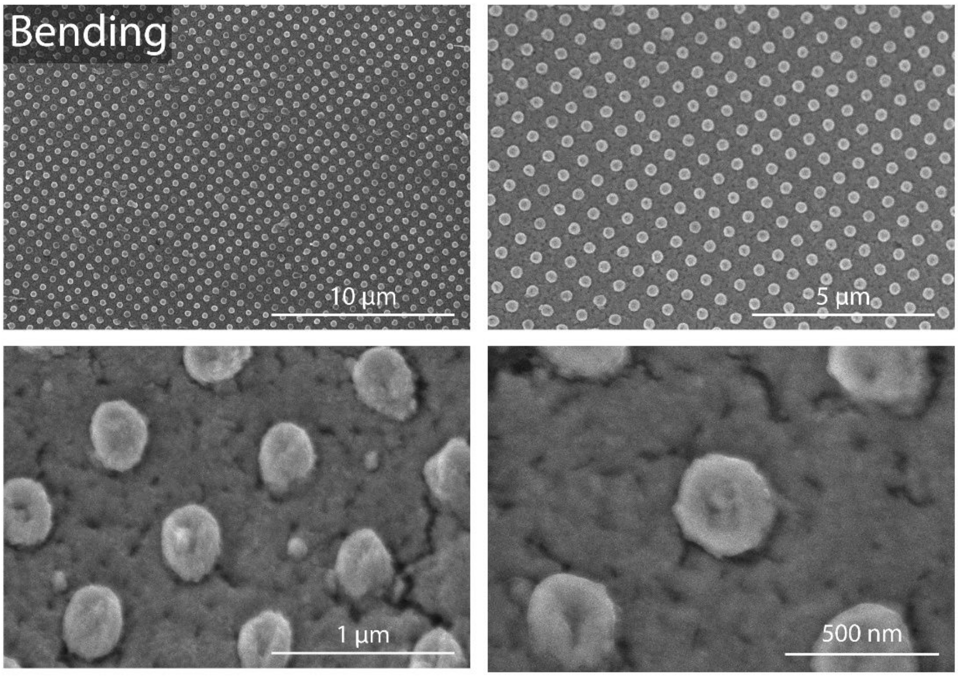

Figure S14. SERS spectra of the Ag-coated pillar CA sample after 50 cycles of bending.

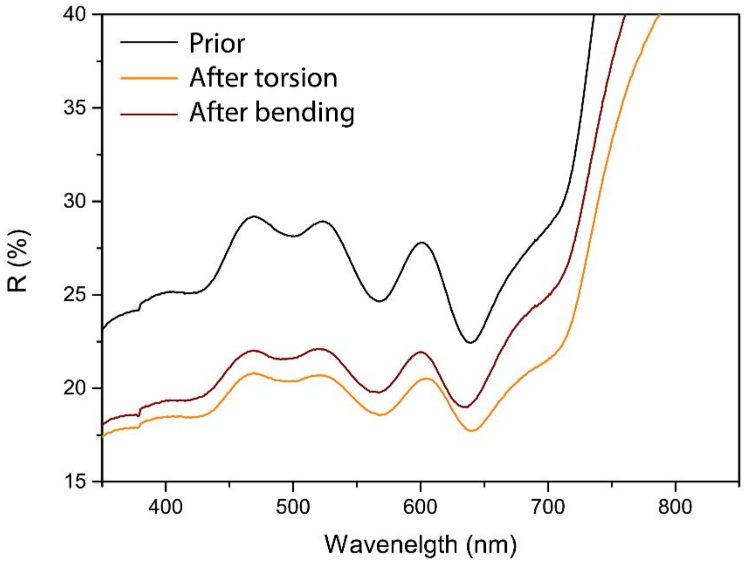

Figure S15. Relative reflectance spectra of the Ag-coated pillar CA sample prior to and after 50 cycles of torsion and bending.

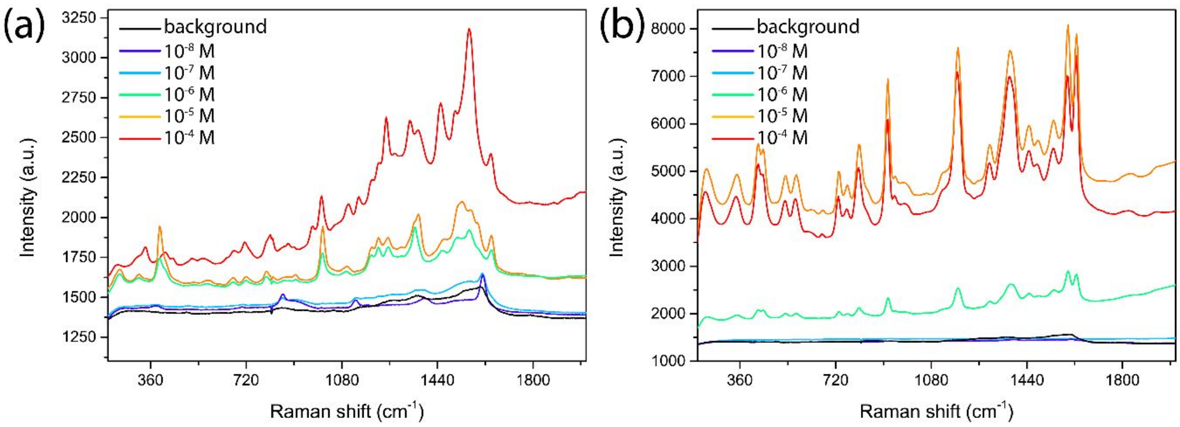

Figure S16. SERS spectra of (a) TMPyP and (b) CV deposited at different concentrations on the Ag-coated pillar CA sample used to determine the detection limit.

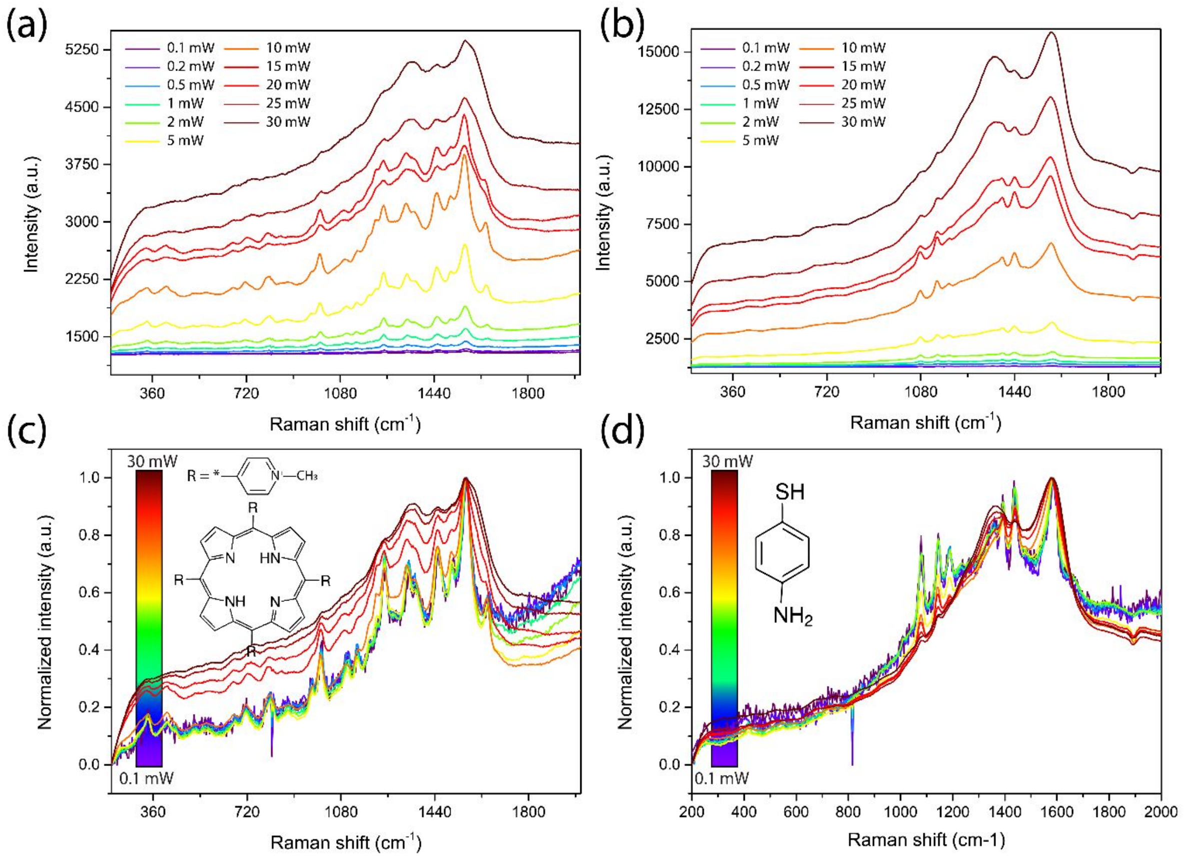

Figure S17. SERS spectra of 10^{-4} M (a) TMPyP and (b) 4-ABT deposited on the Ag-coated pillar CA sample obtained using different powers of the excitation laser. (c) and (d) show the normalized spectra for TMPyP and 4-ABT respectively.
